# The prenatal ultrasonographic detection of myelomeningocele in patients referred to Children's Hospital Medical Center: a cross sectional study

**DOI:** 10.1186/1742-4755-3-6

**Published:** 2006-07-18

**Authors:** Syed Shuja Kazmi, Farideh Nejat, Parvin Tajik, Hadi Roozbeh

**Affiliations:** 1 Department of Neurosurgery, Children's Hospital Medical Center, Tehran University of Medical Sciences, Tehran, Iran; 2 Department of Epidemiology and Biostatistics, School of Public Health, Tehran University of Medical Sciences, Tehran, Iran; 3 Department of Neurosurgery, Sina Hospital, Tehran University of Medical Sciences, Tehran, Iran

## Abstract

**Background:**

To find out about the prenatal diagnosis rate of myelomeningocele (MMC) by ultrasound scan in patients referred to the Children's Hospital Medical Center in Tehran, Iran from July 2004 to July 2005.

**Methods:**

We included 140 children born with MMC and who were referred for management, surgery and treatment of complications associated with it. The ultrasound reports were examined. Data on sex, age, location of MMC, time of prenatal ultrasound and the trimester in which the diagnosis was made along with the results of the diagnosis (MMC, hydrocephalus, or both), were collected.

**Results:**

Among the studied patients, 136 (97.1%) cases had prenatal ultrasound, amongst those, 58 (42.6%) sonographic evaluations were diagnostic for hydrocephalus and/or MMC. The prenatal ultrasound was positive for MMC in 16 (11.8%), hydrocephalus in 25 (18.4%) and both MMC and hydrocephalus in 17 (12.5%) cases. Among all cases with prenatal diagnosis of MMC, 3.4% were detected in the first, 31% in the second and 65.5% in the third trimester. Thoracic/thoracolumbar lesions were found prenatally in 40% of cases, which is significantly higher than the detection rate of other locations including cervical/cervicothoracic and lumbar/lumbosacral/sacral regions diagnosed only in 0% and 21% of cases respectively.

**Conclusion:**

There is a large difference between the detection rate of our population (24.3%) compared to others (68%). Pregnant women should have an ultrasound at 20–22 week for detection of congenital anomalies including MMC.

## Background

Myelomeningocele is a major congenital anomaly caused by a defective closure of the neural tube between the 18–25^th ^days of gestation, resulting in the protrusion of the cord and the meninges through the defective bony encasement of the spinal column [1]. It is a prevalent and crippling defect of the central nervous system (CNS) for which no definitive cure is available. Patients born with MMC have lifelong disabilities including motory, sensory, orthopedic and urologic problems. In spite of being a common birth defect with significant lifelong complications, little progress has been made in the postnatal surgical management of the defects or its associated complications.

During the past decades new screening methods and diagnostic tests have been introduced, allowing an earlier and more accurate diagnosis of fetal anomalies, including MMC [2]. As no definite treatment is available for MMC, the prenatal detection of this handicapping anomaly would help in the timely pregnancy termination as done in developed countries [3]. Studies have shown that the incidence of this malformation is relatively high in Iran, 1.6 per 1000 live births [4], and our experience with MMC patients revealed that most of them were not detected prenatally. Early detection of abnormalities is helpful in making decisions earlier in pregnancy. We retrospectively studied the prenatal ultrasound scan detection rate in patients with MMC referred to our center.

## Methods

The study comprised of 140 patients referred for MMC evaluation, surgery and management of its related complications, to the neurosurgery clinic of Children's Hospital Medical Center in Tehran, Iran from July 2004 to July 2005. This is a referral center that receives most myelomeningocele cases born in Tehran and nearby cities. We registered the sex, age, location of MMC (cervical, cervicothoracic, thoracic, thoracolumbar, lumbar, lumbosacral, and sacral), the time of the prenatal ultrasound scan (according to the trimester), and the trimester in which the diagnosis was made (MMC, hydrocephalus or both) of all patients. The data were analyzed using SPSS software (SPSS Inc., Chicago, IL).

## Results

Among the studied patients, 73 (52.1%) were male, 66 (47.1%) were female and one (0.7%) had an ambiguous genitalia. The age of the patients ranged from one day to five years (median 15 days). The most common location of MMC was thoracolumbar and further distal to it (91.4%). All, except four patients (2.9%) had at least one ultrasound scan during the pregnancy. Ultrasound was done in the first trimester in 55 (39.3%), second trimester in 103 (73.6%) and third trimester in 120 (85.7%) patients. The prenatal scan report was normal in 78 patients (57.4%) and for just 17 of them (12.5%) both, hydrocephalus and MMC, was reported. In 16 patients (11.8%) only MMC, and in 25 (18.4%) only hydrocephalus was detected. MMC was diagnosed in only 33 ultrasonographic evaluations (24.3%). We categorized the lesions into 3 groups according to the MMC locations: cervical/cervicothoracic, thoracic/thoracolumbar and lumbar/lumbosacral/sacral. Detection rates were highest for lesions located in the thoracic/thoracolumbar area (12 of 30; 40%), followed by lesions of the lumbar/lumbosacral/sacral regions (21 of 100; 21%). None of the cervical/cervicothoracic lesions were detected by prenatal ultrasound. The data of prenatal ultrasound scan MMC reports according to the detection time and level of lesion are shown in table 1.

**Table 1:** Frequency of prenatal ultrasound MMC diagnosis according to the detection time and the level of lesion.

**Level of MMC**	**Diagnosed**			**Undiagnosed**	**Total**
			
	1^st ^trimester n (%)	2^nd ^trimester n (%)	3^rd ^trimester n (%)	n (%)	n (%)
Cervical/Cervicothoracic	0 (0)	0 (0)	0 (0)	5 (100)	5 (100)
Thoracic/Thoracolumbar	2 (6.6)	2 (6.6)	8 (26.8)	18 (60)	30 (100)
Lumbar/Lumbosacral/Sacral	0 (0)	8 (8)	13 (13)	79 (79)	100 (100)

**Total**	2 (1.4)	10 (7.4)	21 (15.5)	102 (75.5)	135*(100)

* One patient was omitted due to double MMC sacs in cervical and sacral regions that could not be placed in this categorization.

## Discussion

The incidence of MMC varies from 0.4 to 1.91 per 1000 live births according to the country, culture and socio-economic status [5] and the reported incidence in Iran is 1.6 per 1000 [4]. In developed countries due to folic acid supplementation and increased prenatal detection rates followed by abortion, the incidence has declined significantly over the past few decades. Even then it still affects one in every 2000 live births in the United States [1].

The significant complications in MMC result from associated hydrocephalus, the Arnold-Chiari II malformation and the spinal cord tethering at the site of surgical repair. Despite early and aggressive intervention, nearly 14% of neonates born with MMC do not survive more than 5 years. Although most patients have an IQ above 80, only half are able to live independently as adults [6]. The psychological, social and financial burden on the family and community is enormous, such that the care of affected individual costs about $250,000 per person per lifetime in the United States [6].

As the incidence of this defect is high in Iran [4], it is necessary to take important steps towards primary and secondary preventive measures. As far as the primary prevention is concerned the preventive effect of folic acid on neural tube defects (NTD) is still widely unknown in Iran and many women are not supplemented with folic acid before or during the first trimester of pregnancy. Most of these women receive folic acid after the first or the second month of gestation when the neural plate has either been formed in its natural way into a neural tube or remained as such. Supplementing folic acid after this period can be of no help. In Iran, there is no national health policy for folic acid supplementation to women in the child-bearing age, before planning for pregnancy or during the first trimester. Antenatal ultrasound has been reported to be an effective tool for detecting NTDs, and in Iran it is usually performed for assessing fetal biometric parameters in most pregnancies. Moreover, major structural abnormalities can be detected by ultrasound examination, depending on the time of ultrasound scan. The recommended time and the number of ultrasound scans vary according to the national consensus, the experience of health care providers and other related factors. In the United Kingdom, from the early 1990's pregnant women are offered serum α-fetoprotein screening for NTD's at 15–18 weeks of gestation and an ultrasound scan at 18–22 weeks [3]. Likewise, in France, three ultrasound scans are usually performed around the 12^th^, 22^nd ^and the 32^nd ^week of gestation. In the USA, there is no policy about the number and timing of ultrasound scans and it is mainly performed for selected patients, such as those with a positive family history, clinical symptoms or abnormal maternal serum α-fetoprotein levels [2]. This practice is somewhat similar to the one currently in place in Iran.

Whatever policy is present, screening with the help of ultrasound scan is mainly based on two-dimensional imaging techniques. Doppler and the three-dimensional ultrasound scans have not clearly demonstrated their superiority over the conventional two-dimensional ultrasound scan; however they may be useful to pinpoint the anatomy of the complex lesions [2]. The first trimester conventional ultrasound can detect the majority of cases of anencephaly, but a significant proportion of abnormalities such as the MMC is only detected in the second trimester. However, recent reports show that a transvaginal scan in the first trimester for high-risk population can be of a greater help [7].

There is a long debate regarding which screening modality should be used as both the serum α-fetoprotein and ultrasound are effective, but some recent studies have shown that there is a trend towards increased use of first and second trimester ultrasound scan in a number of developing countries [7,8]. The sensitivity differs, where the gestation, time allowed for scan, quality of equipment, training and skills of operators may affect the prenatal detection rates as well as different scan and biochemical screening policies.

Less severe forms of MMC are compatible with life and early ultrasound diagnosis allows for decision making earlier in pregnancy [3]. Ultrasound in the second trimester can detect MMC by using the standard biparietal cross-sectional view of the fetal head and visualizing the lateral ventricles: the "lemon sign" denotes the scalloping frontal bones, predictive of spina bifida, further, the "banana sign" refers to the abnormally shaped midbrain and an elongated cerebellum in the Arnold-Chiari malformation. Among fetuses with MMC, the "lemon sign" can be detected in 80% and the "banana sign" in 93% of cases. Thus, prenatal finding of an abnormal posterior fossa or ventriculomegaly should prompt the sonographist to rule out an accompanying spinal deformation and serial scans of the fetal spine, performed in a longitudinal and transversal axis as well as parallel to the skin, should be done. An abnormal position of the fetal foot or an enlarged bladder can be the consequence of severe functional impairments, but their absence cannot be considered as reassuring [5].

Obesity, oligohydramnios or poor fetal position can cause an inability to obtain adequate images. At the same time ultra-fast Magnetic Resonance Imaging (MRI) provides detailed, reproducible images of fetal anomalies but it is difficult to adequately evaluate the fetus before 18 weeks of gestation because of its small size. MRI has been helpful to augment the ultrasound diagnosis of CNS abnormalities and is less affected by the above-mentioned pitfalls of ultrasound [9].

Other studies have reported different MMC detection rates. In a large study across Europe, the range varied from 33% to 100% in different countries, with a mean of 68% [3]. Our detection rate (24.3%) was much lower, which can be due to lack of a national screening policy for MMC, not paying enough attention to find MMC in routine ultrasound and low resolution ultrasound equipment. We suggest referring all pregnant women at 20–22 weeks of gestation to a well equipped ultrasound center with qualified operators for a formal search of fetal anomalies, including MMC.

## Conclusion

Due to the lack of a national policy for prenatal ultrasound screening in Iran, the ultrasound scans performed during pregnancy are either according to the protocol of the health center, the health care provider or solely on the woman's request. Measurement of maternal serum α-fetoprotein at 15–18 weeks and a detailed high resolution ultrasound scan at 20–22 weeks of gestation can help in the early detection of MMC. The law in Iran allows abortion only up-to the fourth month of gestation. As it is difficult to diagnose MMC in the first trimester, the delay for pregnancy termination in Iran for this rather prevalent anomaly should be increased up to the sixth month of gestation.

## Abbreviations

CNS: Central Nervous System

MMC: Myelomeningocele

NTD: Neural Tube Defect

MRI: Magnetic Resonance Imaging

## Competing interests

The author(s) declare that they have no competing interests.

## Authors' contributions

The authors contributed equally to this work.
